# Hearing and communicative skills in the first years of life in children with congenital Zika syndrome

**DOI:** 10.1016/j.bjorl.2020.05.007

**Published:** 2020-06-11

**Authors:** Lucianna Cabral de Almeida, Lílian Ferreira Muniz, Rebeka Jacques Maciel, Danielle Seabra Ramos, Kátia Maria Gomes de Albuquerque, Ângela Maria Carneiro Leão, Matheus Vota de Mendonça, Mariana de Carvalho Leal

**Affiliations:** a Universidade Federal de Pernambuco (UFPE), Recife, PE, Brazil; b Universidade Católica de Pernambuco (UNICAP), Recife, PE, Brazil; c Hospital Agamenon Magalhães, Recife, PE, Brazil

**Keywords:** Zika virus infection, Hearing disorders, Child development

## Abstract

**Introduction:**

Microcephaly is recognized as one of the main consequences of congenital Zika syndrome, but other serious problems such as global hypertonia, irritability, excessive crying, swallowing disorders, seizures, visual impairment and sensorineural hearing loss have been identified as associated with the syndrome.

**Objective:**

Describe the developmental characteristics of hearing and language skills in the first year of life of children with normal hearing thresholds’ and congenital Zika syndrome.

**Methods:**

This is a cross-sectional study that evaluated hearing and language skills in the first year of life of 88 children with normal peripheral hearing and confirmed congenital Zika syndrome. All children were submitted to a behavioral auditory test and a validated questionnaire addressed to parents or caregivers, which was used as an instrument for assessing hearing and communicative skills.

**Results:**

The delay in communicative skills was present in 87.5% of the children, while 44.3% of them demonstrated a delay in hearing acuity. Only the alteration of cervical motor control presented as a statistically significant association with delays in both skills (*p*-value = 0.006 and <0.001 for hearing and communicative skills, respectively), while the presence of microcephaly and the degree of its severity were only associated with delayed development of communicative skills.

**Conclusion:**

Despite a normal peripheral auditory system, children with congenital Zika syndrome may demonstrate delayed language development by having neurological damage at the center of auditory processing, requiring more specific studies to clarify language acquisition in this population.

## Introduction

The Zika Virus (zikV), an arbovirus of the Flaviviridae family, was first isolated in humans in 1952, and related only to sporadic mild infections until 2007, when a Zika fever epidemic occurred on Yap Island, Micronesia, and was considered an emerging virus.^1^, ^2^

Viral transmission occurs through insect bites, especially the Aedes aegypti, along with Aedes albopictus. In addition, zikV can be transmitted through blood transfusion and through sexual intercourse, although the infectivity potential of the latter is not fully understood.^3^

In 2013, a major epidemic was reported in French Polynesia, while the indigenous circulation of zikV in the Americas was confirmed for the first time only in February 2014. But it was only by December 2014 that the zikV gained notoriety from an epidemic of unknown exanthematous disease in Brazil, later identified as zikV infection and associated with congenital microcephaly cases.^3^ The neonates affected in the Brazilian microcephaly epidemic had skull imaging exams with findings suggestive of congenital infection such as brain calcifications, ventriculomegaly and cortical atrophy, and the confirmation of the relationship between the zika virus and neonatal microcephaly occurred when viral genetic material was detected by Polymerase Chain Reaction with Reverse Transcriptase (RT-PCR) in samples of amniotic fluid from two pregnant women whose fetuses were diagnosed with microcephaly by ultrasound exams.^3^

Microcephaly is recognized as one of the main consequences of CZS, presenting with varying degrees of severity. Children with head circumference 3 standard deviations below that expected for sex and gestational age are recognized as severely affected and present with significant changes in cognitive capacity, while children with non-severe microcephaly, head circumference of one or two standard deviations below that expected for sex and gestational age probably show mild language delays, mild cognitive impairment and even normal cognitive development. Furthermore, other characteristics of the syndrome may contribute to increase the clinical repercussions on neuropsychomotor development throughout the lives of these children, such as dysphagia, seizures, visual impairment and hearing loss. The latter seems to be present in about 5.8% as presented in a cross-sectional study with seventy children confirmed with congenital zika infection, mostly associated with severe microcephaly.^4^, ^5^, ^6^, ^7^, ^8^

Nevertheless, in view of the multifactorial impairment in the neurodevelopment of children with CZS, it is possible that children with this syndrome have delayed language development even in conditions of normal peripheral hearing. Thus, the present study aimed to describe the characteristics of the auditory and communication development skills in the first year of life among children with normal hearing and CZS.

## Methods

This is a cross-sectional descriptive study that evaluated hearing and language skills in the first year of life of 88 children with normal hearing and congenital zika syndrome referred to Agamenon Magalhães Hospital, a tertiary health care center in Pernambuco – Brazil.

This article came as part of the work from a study group that monitored children born at the peak of the microcephaly related to Zika virus outbreak in Brazil, especially at Pernambuco state, that started in November 2015. In a previous study, we evaluated 69 children with a confirmed diagnosis of CZS, finding 4/69 (5.8%) of hearing loss in this population. The population evaluated in this study consists of 65 children from the previous study and 23 others who were included later with normal click ABR at 35 dB NHL. Children who had sensorineural hearing loss in the previous research were excluded, since the aim of this study was to assess the auditory and communication skills that could be related to the central impairment of CSZ. In this situation, with normal peripheral auditory input, we did not expect to find delay in these skills assessed.

The study was approved by the Research Ethics Committee of the former hospital under the number 1.472.742. All the children were previously diagnosed with CZS through the presence of typical radiologic patterns from skull Computerized Tomography (CT) or Magnetic Resonance Imaging (MRI) associated with laboratory confirmation of Zika virus infection by a positive Zika virus-specific Immunoglobulin M (IgM) capture Enzyme-Linked Immuno-Sorbent Assay (ELISA) performed on cerebrospinal fluid.^8^ Other infectious causes of congenital sensorineural hearing loss, including CMV, toxoplasmosis, herpes simplex, and syphilis were excluded by serologic testing of infants and their mothers. The click ABR results was used as an inclusion criteria, so that only children with presumably normal hearing, according to the criteria of the Joint Committee on Infant Hearing (JCIH),^9^ could participate in this study.

The presence and timing of the rash during pregnancy (first, second or third trimesters), presence and severity of microcephaly (non-severe microcephaly when head circumference at birth was less than 2 standard deviations from the mean for gestational age and gender, while severe microcephaly when head circumference at birth was less than 3 standard deviations from the mean for gestational age and sex) and the presence or absence of cervical motor control was observed.

A validated questionnaire addressed to parents or caregivers was used as a tool for assessing hearing and communication skills, which included three specific questions for the development of these skills in each month of the child's life during the first year of life (Appendix 1).^6^ Children were also subjected to a behavioral hearing test through the use of sound stimuli emitted by musical instruments (rattle, drums and agogô) and Ling sounds, in order to assess the detection of vowel phonemes and consonants.^10^ The stimuli were presented a half meter away from the ear using cardinal positions, under and above the child. All presentations were performed at low intensity, inside an acoustic booth, in the presence of two audiologists, positioned one behind (sending the stimulus) and one in front of the child (diverting the child's attention from another examiner and observed the reactions). The child was positioned on the mother's lap with the back resting on her but leaving the head free to move. Response was considered when child demonstrated the same behavior at least twice for same stimuli consecutively.

For analysis of the results, the responses were compared to the markers of hearing and language development, according to Northern & Downs.^7^ When analyzing the questionnaire it was considered a delay in hearing or communicative skills, if presenting at least a one “no” answer to the questions related to each skill indicated for the age of the children.

Data were analyzed using Pearson's Chi-Square test or Fisher's exact test and the margin of error used was 5%.

## Results

We evaluated 36 female children and 52 male children. Most children had microcephaly (71.6%), especially in the severe form, 47.7%. The skin rash was reported by 76% (67 women) of the mothers, with 53.4% in the first trimester of gestation. The lack of cervical motor control was present in 80% (Table 1), despite the mean age of the children of 11.4 months.

**Table 1:** Epidemiological characteristics of children with congenital Zika virus syndrome.

Characteristic (number with information available)	*n*	%
*Total*	88	100.0

*Sex*		
Female	36	40.9
Male	52	59.1

*Age (months)*		
Mean	11.4	
Range	6–12	

*Microcephaly*		
With microcephaly	63	71.6
Without microcephaly	7	8.0

*Degree of microcephaly*		
Non-severe microcephaly	21	23.9
Severe microcephaly	42	47.7

*Timing of rash during pregnancy*		
First trimester	47	53.4
Second trimester	15	17.0
Third trimester	5	5.7

*Motor cervical control*		
Normal	17	19.3
Abnormal	71	80.7

Concerning the behavioral hearing tests, 42 children were tested and 41 (97.6%) presented auditory attention to instruments, 37 (88.10%) presented attention to Lings sounds, but only 11 (88.1%) showed normal sound localization (Table 2). However, the sound location presented a statistically significant association with cervical motor control (Table 3).

**Table 2:** Result of the behavioral audiological test of children with congenital Zika virus syndrome.

	Normal, *n* (%)	Abnormal, *n* (%)	Total, *n* (%)
Attention to sound	41 (97.62)	21 (2.38)	42 (100)
Sound source localization	11 (26.20)	31 (73.80)	42 (100)
Ling's sound detection	37 (88.10)	5 (11.90)	42 (100)

**Table 3:** Assessment of the location of the sound source and cervical motor control of children with congenital Zika virus syndrome.

Cervical motor control	Sound source localization						*p*-value
	Normal		Abnormal		Total		
	*n*	%	*n*	%	*n*	%	
Cervical motor control	6	54.54	5	45.46	11	100.0	*p*^a^ < 0.020^b^
No cervical motor control	5	16.13	26	83.87	31	100.0	
Total	11	85.7	31	14.3	42	100.0	

a Fisher's exact test.

b Statiscally meanfull association.

The delay in communicative skills was present in 87.5% of the children and 44.3% of them demonstrated a delay in hearing skills, considering questionnaire results. Only the alteration of cervical motor control presented as a statistically significant association with both the delays of hearing and communicative skills (*p* = 0.006 and *p* < 0.001, respectively), while the presence of microcephaly and the degree of its severity were associated with delayed development of communicative skills (Table 4, Table 5).

**Table 4:** Evaluation of hearing skills of children with congenital Zika virus syndrome.

Characteristic	Hearing skills in children						*p*-value
	Delayed		Normal		Total		
	*n*	%	*n*	%	*n*	%	
*Microcephaly*							*p*^a^ = 0.452
With microcephaly	29	16.0	34	51.0	63	100.0	
Without microcephaly	2	28.6	5	71.4	7	100.0	
Total	31	44.3	39	55.7	70	100.0	

*Degree of microcephaly*							*p*^b^ = 0.371
Severe microcephaly	21	50.0	21	50.0	42	100.0	
Non-severe microcephaly	8	38.1	13	61.9	21	100.0	
Total	31	44.3	39	55.7	70	100.0	

*Timing of rash during pregnancy*							*p*^a^ = 0.403
First trimester	25	53.2	22	46.8	47	100.0	
Second trimester	5	33.3	10	66.7	15	100.0	
Third trimester	2	40.0	3	60.0	5	100.0	
Total	32	47.8	35	52.2	67	100.0	

*Motor cervical control*							*p*^b^ = 0.006
Normal	3	17.6	14	82.4	17	100.0	
Abnormal	39	54.9	32	45.1	71	100.0	
Total	42	47.7	46	52.3	88	100.0	

a Exact Fisher's test.

b Pearson's Chi-Square test.

**Table 5:** Evaluation of communicative skills of children with congenital Zika virus syndrome.

Characteristic	Language skills in children						*p*-value
	Delayed		Normal		Total		
	*n*	%	*n*	%	*n*	%	
*Microcephaly*							*p*^a^ < 0.001^b^
With microcephaly	58	92.1	5	7.0	63	100.0	
Without microcephaly	2	28.6	5	71.4	7	100.0	
Total	60	85.7	10	14.3	70	100.0	

*Degree of microcephaly*							*p*^a^ = 0.039^b^
Severe microcephaly	41	97.6	1	2.4	42	100.0	
Non-severe microcephaly	17	81.0	4	19.0	21	100.0	
Total	60	85.7	10	14.3	70	100.0	

*Age at birth*							*p*^a^ = 0.128
Term	64	90.1	7	9.9	71	100.0	
Preterm	8	72.7	3	27.3	11	100.0	
Total	72	87.8	10	12.2	82	100.0	

*Timing of rash during pregnancy*							*p*^a^ = 1.000
First trimester	42	89.4	5	10.6	47	100.0	
Second trimester	14	93.3	1	6.7	15	100.0	
Third trimester	5	100.0	–	–	5	100.0	
Total	61	91.0	6	9.0	67	100.0	

*Motor cervical control*							*p*^a^ < 0.001^b^
Normal	8	47.1	9	52.9	17	100.0	
Abnormal	69	97.2	2	2.8	71	100.0	
Total	77	87.5	11	12.5	88	100.0	

a Exact Fisher's test.

b Statistically meaningful association.

## Discussion

The severe impairment of cortical development, with malformation and volumetric reduction of the parenchyma, in addition to cortical and subcortical calcifications, hypomyelination or demyelination of white matter and ventriculomegaly are the main characteristics of the neurological impairment in CZS, and are related to zikV viral neurotropism mainly in congenital infections in the first months of pregnancy, which supports the disruptive type of anomalous brain development found in these children.^11^

This immaturity of the central nervous system could justify the large number of children with delayed communication and auditory skills (87.5%), since language and hearing development is a product of functional peripheral hearing, neurological maturation and cognitive load.^7^, ^12^

According to Northern and Downs, from 4 to 6 months of age the child can already turn his head toward the sound source and, from 7 months, the neck muscles already allow a direct location, but despite the average age of 11.4 months among the children studied, most did not have cervical motor control. This neck hypotony was statistically associated with a greater delay in hearing and communication skills, which can represent the delay in neuropsychomotor development that occurs in CZS and makes it difficult to search for the sound source and, consequently, influences children's reactions to sounds.

In this study we also observed an important relation between cervical motor skills and sound localization skill. Even if the majority of the group were capable of showing attention to some sound, they were not able to localize sound as was expected for their age. Attention to sound requires a passive reaction and on the other hand localization requires an active response and normal function of cervical motor skills, which we do not see in this group.

The integrity and effective functioning of hearing skills are prerequisites for language acquisition and development. In order for the child to be able to recognize and understand speech, he must therefore be able to pay attention, detect, discriminate and locate sounds, in addition to memorizing and integrating auditory experiences.

Flor, in her study, evaluated 22 children with CZS and microcephaly, and observed a delay in the four fields of the Denver scale for neuropsychomotor development.^13^, ^14^ Moreover a study with premature children showed that despite all children having passed hearing screening tests, there was a high rate of disorder in the acquisition of language skills, suggesting the relationship between delayed neuropsychomotor development and language acquisition.^15^

At around 6 months of age, normally developing children are expected to produce babbling associated with adult facial expressions and speech imitations, and at 12 months, about 50% of children will produce their first words, although it was based on a questionnaire addressed to parents, these language precursors were absent at the expected age in 87.5% of the population in the present study, especially in children with severe microcephaly. We emphasize, therefore, the relevance of reporting aspects of the development of children with CZS who, despite having normal peripheral hearing, are markedly delayed in the acquisition of language precursors.

Identifying and understanding possible changes in the peripheral and central auditory system in early childhood is crucial for the development of early intervention strategies, since the first three years of life are known as a critical period for the emergence and maturation of brain synapses, a process called neuronal plasticity.^6^, ^7^ Thus, despite being a cross-sectional study with low analytical power, our results call attention to an important impairment of communicative skills in children with CZS even with normal peripheral hearing.

## Conclusion

We believe that neurological damage to the auditory pathway and auditory cortex, as well as neuropsychomotor impairment, can contribute to poor language development, as seen in the respective associations between severity of microcephaly and delayed communication skills and between lack of adequate cervical motor control and delay in auditory and communicative skills. Thus, longitudinal studies are necessary to better understand language development in children with CZS, even in those with normal peripheral hearing.

## Conflicts of interest

The authors declare no conflicts of interest.

## References

[1] Duffy M.R., Chen T.-H., Hancock W.T., Powers A.M., Kool J.L., Lanciotti R.S. (2009). Zika virus outbreak on Yap Island, Federated States of Micronesia. N Engl J Med.

[2] Terskikh A.V., Pinto A., Farhy C., Gorshkov K., Strongin A.Y., Huang C.-T. (2019). Zika virus: origins, pathological action, and treatment strategies. Front Microbiol.

[3] Lowe R., Barcellos C., Brasil P., Cruz O.G., Honório N.A., Kuper H. (2018). The zika virus epidemic in brazil: From discovery to future implications. Int J Environ Res Public Health.

[4] Eickmann S.H., Carvalho M.D.C.G., Ramos R.C.F., Rocha MÂW, Linden V. van der, Silva P.F.S.da. (2016). Síndrome da infecção congênita pelo vírus Zika TT - Zika virus congenital syndrome TT – Síndrome de la infección congénita del virus Zika. Cad Saude Publica (Online).

[5] Ramos R.C.F., Rodrigues L.C., Van Der Linden V., Ferreira T.S.A., Neto S.S.C., Almeida L.C. (2016). Hearing loss in infants with microcephaly and evidence of congenital zika virus infection – Brazil, November 2015–May 2016. MMWR Morb Mortal Wkly Rep.

[6] Araújo E.S., Lima F.S., Alvarenga K.F. (2013). Monitoramento de crianças com indicadores de risco para a deficiência auditiva. Rev CEFAC.

[7] Northern J.L., Downs M.P. (2005).

[8] Cordeiro M.T., Pena L.J., Brito C.A., Gil L.H., Marques E.T. (2016). Positive IgM for Zika virus in the cerebrospinal fluid of 30 neonates with microcephaly in Brazil. Lancet.

[9] Cherow E. (2000). Year 2000 position statement: principles and guidelines for early hearing detection and intervention programs. Am J Audiol.

[10] Agung K.B., Purdy S.C., Kitamura C. (2005). The ling sound test revisited. Aust New Zeal J Audiol.

[11] Hazin N., Poreti A., Martelli C.M.T., Huisman A.T. (2016). Computed tomographic findings in microcephaly associated with zika virus. N Engl J Med.

[12] Villa Flor C.J.D.R., Guerreiro C.F., Dos Anjos J.L.M. (2017). Desenvolvimento neuropsicomotor em crianças com microcefalia associado ao zika vírus. Rev Pesqui Fisioter.

[13] (2016).

[14] Barbosa C., Luiz L., Garcia M.V., Perissinoto J., Goulart A.L., Azevedo M.F.D. (2016). Relation between auditory abilities in the first year of life and language diagnosis in pre-terms. Rev CEFAC.

[15] Fernandes D.M.Z., Lima M.C.M.P., Gonçalves V.M.G., Françozo MDFD.C. (2008). Follow-up of language development in infants with risk factors for hearing loss. Rev Soc Bras Fonoaudiol.

