# Supplementary figures and images for: Hearing and communicative skills in the first years of life in children with congenital Zika syndrome
Source: Braz J Otorhinolaryngol. 2020 Jun 11;88(1):112–7. doi: 10.1016/j.bjorl.2020.05.007 (PMC9422677; doi:10.1016/j.bjorl.2020.05.007)

**Appendix 1** Language and auditory development inventory – adapted from Alvarenga et al.


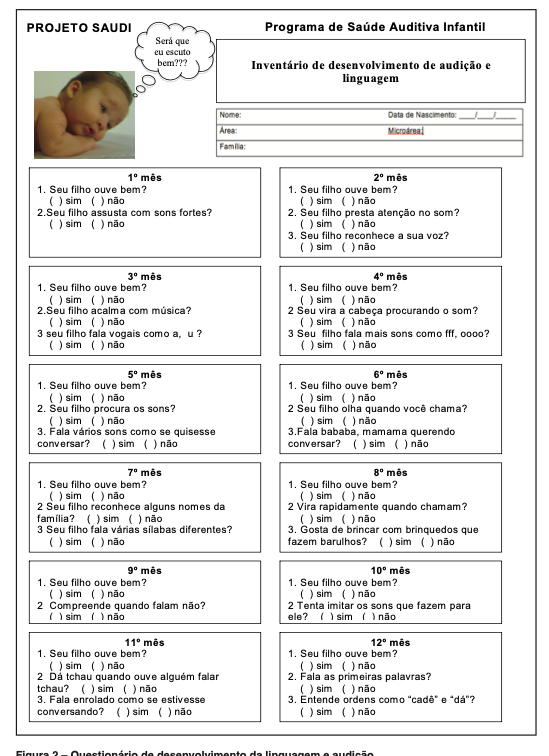

Supplement: Supplementary file 1 [file mmc1.docx]
